# Epidemiological, clinical, and molecular analysis of human adenovirus infections in hospitalized children with acute respiratory infections in Tianjin, China

**DOI:** 10.3389/fcimb.2025.1600990

**Published:** 2025-07-14

**Authors:** Yulian Fang, Min Lei, Lu Zhang, Mengzhu Hou, Ning Wang, Chunquan Cai

**Affiliations:** ^1^ Department of Pediatric Research Institute, Children’s Hospital of Tianjin University/Tianjin Children’s Hospital, Tianjin, China; ^2^ Tianjin Key Laboratory of Birth Defects for Prevention and Treatment, Children's Hospital of Tianjin University/Tianjin Children's Hospital, Tianjin, China; ^3^ Department of Respiratory, Children’s Hospital of Tianjin University/Tianjin Children’s Hospital, Tianjin, China

**Keywords:** human adenovirus, acute respiratory infections, children, epidemiology, molecular types

## Abstract

**Background:**

Human Adenovirus (HAdV) is a significant pathogen for acute respiratory infections(ARIs) in children. However, its epidemiological patterns, serotype distribution changes, and molecular mechanisms associated with severe pneumonia during and after the COVID-19 pandemic require further elucidation through large-scale and molecular typing studies.

**Methods:**

This study used a retrospective cohort design to analyze 28060 respiratory specimens from Tianjin Children’s Hospital from March 2022 to March 2024. HAdV detection and typing were performed through targeted high-throughput sequencing and PCR-based amplification of Penton, Hexon, and Fiber genes for phylogenetic analysis. Additionally, clinical data were compared to assess differences in clinical presentations among pediatric patients infected with different HAdV types.

**Results:**

The overall HAdV detection rate was 8.9% (2,484/28,060), with significant male predominance (9.2% *vs*. 8.4%, *P* = 0.019) and age-specific susceptibility peaking in school-aged children (10.4%, *P* < 0.001). Seasonal patterns demonstrated winter predominance (15.9%), contrasting with other seasons (*P* < 0.001). Genotyping of 1,914 positive specimens demonstrated HAdV-3 dominance (53.4%, 1,022), followed by HAdV-7 (17.7%, 338), HAdV-2 (8.4%, 160), HAdV-1 (7.9%, 152), and HAdV-21 (6.4%, 122). The diagnosis mainly included pneumonia, bronchitis, adenopharyngitis, and upper respiratory tract infections (URTIs). Genotype-clinical correlations showed distinct patterns: HAdV-3 (55.6%) and HAdV-7 (20.9%) predominated in pneumonia cases, with HAdV-7 linked to severe pneumonia (*P*<0.001). HAdV-3 (40.6%) and HAdV-2 (16.7%) were more common in adenopharyngitis, while HAdV-3 and HAdV-21 were more common in bronchitis (51.2% and 11.1%) and URTIs (31.9% and 19.1%). Molecular characterization revealed structural conservation in the Penton protein of HAdV-C and identified Hexon as the most polymorphic region with 85 variable sites, indicating divergent evolutionary pressures across viral domains.

**Conclusion:**

HAdV-3, HAdV-7, HAdV-2, and HAdV-1 were the predominant HAdV types in children hospitalized with ARIs in Tianjin. Moreover, not only the epidemiological characteristics of different HAdV types vary, but there are also certain differences in the clinical symptoms and outcomes of children infected with different types of HAdV. Therefore, it is essential to differentiate HAdV types for epidemiological surveillance and clinical management purposes.

## 1 Introduction

Human adenovirus (HAdV), a non-enveloped dsDNA virus of the genus *Mastadenovirus* (family Adenoviridae), represents a significant global pathogen causing heterogeneous clinical manifestations across age groups. Pediatric populations exhibit heightened susceptibility, with 80% of infections occurring in children under 4 years of age due to waning maternal antibodies and immature adaptive immunity (Lynch and Kajon, 2016). The International Committee on Taxonomy of Viruses (https://talk.ictvonline.org/taxonomy) classifies more than 120 genotypes into seven species (A-G), with distinct tissue tropisms dictating clinical outcomes. Acute respiratory infections (ARIs) are commonly linked to species B (B3, B7, B14, B16, B21), C (C1, C2, C5, C6, C57), and E (E4) (Lynch and Kajon, 2016; Tian et al., 2021), manifested as pneumonia, bronchitis, upper respiratory tract infections (URTIs), or tonsillitis (Wen et al., 2021). Notably, approximately one-third of HAdV-associated pneumonia progresses to severe adenoviral pneumonia, characterized by radiographic consolidation and extrapulmonary complications (Lu et al., 2013; Wang et al., 2021). Despite this clinical burden, evidence remains limited regarding genotype-specific virulence determinants. This emphasizes the importance of rapid and accurate HAdV genotyping in clinical diagnosis and epidemiological investigation.

Conventional HAdV serotype determination relies on neutralization assays and hemagglutination inhibition tests, which are time-consuming and reagent-limited. The emergence of novel recombinant strains through homologous recombination complicates traditional serological typing, as evidenced by circulating variants containing discordant antigenic determinants. Modern molecular approaches, particularly multiplex PCR and real-time quantitative PCR (qPCR), demonstrate superior diagnostic utility with higher sensitivity for established genotypes (Ison and Hayden, 2016). However, partial genome sequencing strategies (e.g., Hexon hypervariable region analysis) carry inherent misidentification risks, as critical recombination breakpoints frequently occur outside targeted loci. To ensure accurate molecular epidemiology, the WHO HAdV Working Group (http://hadvwg.gmu.edu) recommends integrated analysis of three capsid genes (Hexon, Penton base, and Fiber) (Wu et al., 2022).

During the last decade, outbreaks of respiratory tract infections caused by HAdV have occurred frequently in many countries including China (Barnadas et al., 2018; Zhao et al., 2020; Huang et al., 2021; Pscheidt et al., 2021; Huang et al., 2023). While genotype-specific virulence has been hypothesized, only a few published pediatric studies incorporate molecular subtyping data, and existing evidence suggests a correlation between genotypes and clinical outcomes (Lin et al., 2017; Wang et al., 2021). Thus, the genotyping of HAdV is crucial for understanding local epidemiology, tracking virulence variants, and guiding vaccine development. Notably, the widespread implementation of non-pharmaceutical interventions (NPI) during the COVID-19 pandemic significantly altered the transmission patterns of respiratory viruses, including HAdV (Huang et al., 2023). In the post-pandemic era, the epidemiological characteristics, predominant serotype distribution, and associations with disease severity of HAdV have exhibited new dynamics, urgently requiring continuous surveillance and in-depth investigation (Yan et al., 2024; Zhao et al., 2024; Contes and Liu, 2025; Han et al., 2025). The aim of this study was to investigate the epidemiological, clinical, and molecular characteristics of HAdV infections among hospitalized children with ARIs in Tianjin from March 2022 to March 2024, aiming to inform future prediction and intervention strategies for HAdV-related diseases. The findings provide scientific evidence for the prevention and control of HAdV-related diseases.

## 2 Materials and methods

### 2.1 Patients and specimens

**Figure 1:**
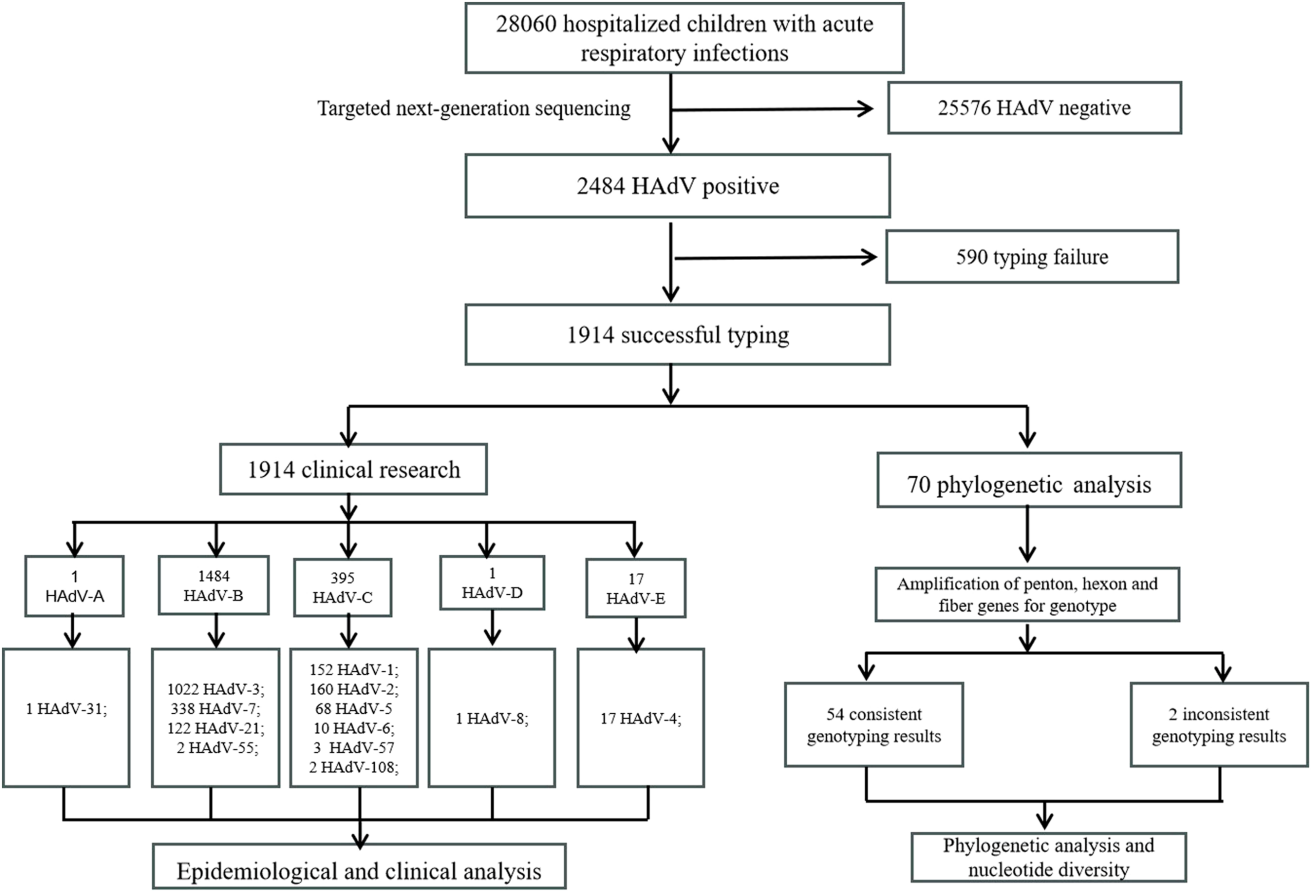
Flow diagram of study design.

This retrospective cohort study was conducted from March 2022 to March 2024 at Tianjin Children’s Hospital in Tianjin. Clinical specimens (nasopharyngeal swabs, sputum, and bronchoalveolar lavage fluid) were collected from children with ARIs within 24 hours after hospitalization. All specimens were stored at -80°C for further genotyping. Demographic and clinical data of HAdV-positive patients by targeted next-generation sequencing were obtained from their medical records. The inclusion criteria were as follows (1): presentation of typical ARIs symptoms (e.g., fever, cough, nasal congestion) (2); hospitalization during the specified period; (3) age range of 1 day to 18 years; (4) informed consent from patients or guardians. Exclusion criteria were as follows: (1) hospital-acquired infections; (2) patients with incomplete electronic medical records; (3) cause of hospitalization other than ARIs. Severe pneumonia was diagnosed according to the Chinese 2019 version of diagnosing and treating children’s community-acquired pneumonia (National Health Commission of the People’s Republic of China and State Administration of Traditional Chinese Medicine, 2019). This study protocol was approved by the ethics committee of the Tianjin Children’s Hospital and conducted by the Declaration of Helsinki guidelines. The parents or guardians of all participants signed informed consent. Specimens for this study were collected in Tianjin, located in the North China region at approximately 39°N latitude in the Northern Hemisphere. The seasons were defined according to the Northern Hemisphere standards: spring (March to May), summer (June to August), autumn (September to November), and winter (December to February of the following year). A flow diagram of the study design is shown in **Figure 1**.

### 2.2 Detection of HAdV and molecular genotyping

HAdVs were detected and genotyped using a multiplex PCR-based targeted next-generation sequencing (tNGS) platform. This assay not only identified HAdV presence but also resolved 13 distinct genotypes (HAdV-1, 2, 3, 4, 5, 6, 7, 8, 21, 31, 55, 57, and 108). The diagnostic panel development and validation were conducted by JinYu Biotechnology. Viral DNA was randomly extracted from 100 HAdV-positive clinical specimens using the QIAamp MinElute Virus Spin Kit (QIAGEN, Germany) following the manufacturer’s protocol. Target regions encompassing the Hexon, Penton base, and Fiber genes were amplified by conventional polymerase chain reaction (PCR) as previously described (Wu et al., 2022). Amplification products were analyzed by electrophoresis on 1.5% agarose gels, and positive amplification products were sequenced by GENEWIZ (Suzhou, China) using Sanger sequencing technology. Obtained sequences were aligned against the National Center for Biotechnology Information (NCBI, https://www.ncbi.nlm.nih.gov/) GenBank database using Basic Local Alignment Search Tool (BLAST). Genotype assignment was determined through maximum sequence identity matching across the Hexon, Penton base, and Fiber. Phylogenetic reconstruction and amino acid substitution analyses were subsequently performed to characterize viral evolution.

### 2.3 Phylogenetic and amino acid mutation analysis

For preliminary genotyping analysis, the 188 successfully sequenced nucleotide fragments were aligned against the NCBI database (https://www.ncbi.nlm.nih.gov). Reference sequences comprising prototype strains with definitive genotypes and circulating strains exhibiting >99% nucleotide identity to study isolates were downloaded from GenBank. Sequences with length <1000 bp were excluded. Multiple sequence alignment was performed using ClustalW, followed by trimming all sequences to uniform length. Maximum likelihood trees were reconstructed in MEGA-X (v10.2.6) with branch support assessed by 1000 bootstrap replicates. Nucleotide and amino acid sequence homology analyses, along with mutational profiling, were performed using BioEdit v7.2.0 (https://www.mbio.ncsu.edu/BioEdit/page2.html). The sequences of the Hexon, Penton base, and Fiber genes characterized in this study have been deposited in the GenBank database under accession number PV106134-PV106159.

### 2.4 Recombination analysis

For strains exhibiting genotypic discordance across the Penton, Hexon, and Fiber genes (indicative of potential recombination), the trimmed nucleotide sequences of these three genes from each suspected recombinant strain were concatenated in-frame. Corresponding gene sequences from Human adenovirus C (HAdV-C) prototype strains and closely related, genotypically well-defined circulating strains retrieved from GenBank were similarly trimmed, aligned with the study sequences, and concatenated. Recombination events within the concatenated sequences were assessed using the RDP5 software package, employing seven detection algorithms: RDP, GENECONV, 3Seq, Chimaera, SiScan, MaxChi, and LARD. Recombination events detected by at least five methods with a p-value < 0.05 were considered statistically supported. Putative recombination breakpoints identified by RDP5 were further characterized and visualized using SimPlot software (version 3.5.1), with the suspected recombinant strain as the query sequence. BootScan analysis was performed using a window size of 200 nucleotides (nt), a step size of 20 nt, 100 replicates, gap stripping enabled, and the Kimura 2-parameter distance model.

### 2.5 Nucleotide diversity analysis

Nucleotide diversity plots were constructed using the DNA Sequence Polymorphism software (DnaSP v5.10.01; www.ub.edu/dnasp/). Sites with alignment gaps were excluded. The analysis was performed with a sliding window length of 100 bps and step size of 25 bps.

### 2.6 Statistical analysis

Statistical analysis was performed using IBM SPSS Statistics (version 23.0). Continuous variables were described as mean ± standard deviation for normally distributed data or median with interquartile range (IQR) for nonparametric distributions. Continuous variables were compared using the *t*-test or the Mann-Whitney U test. Categorical data were displayed as numbers and percentages, and were compared by chi-square test or Fisher’s exact test, as appropriate. Each statistical test was two-sided, and *P* values of <0.05 were considered statistically significant.

## 3 Results

### 3.1 Characteristics of inpatient children with ARIs

Between March 2022 and March 2024, a total of 28,060 pediatric patients were hospitalized with ARIs. The cohort comprised 15,584 males (55.5%) and 12,476 females (44.5%), yielding a male-to-female ratio of 1.25:1. Notably, 45.1% of cases (12,655/28,060) involved children under 3 years of age.

### 3.2 Epidemiological characteristics of HAdV infection in children with ARIs

**Figure 2:**
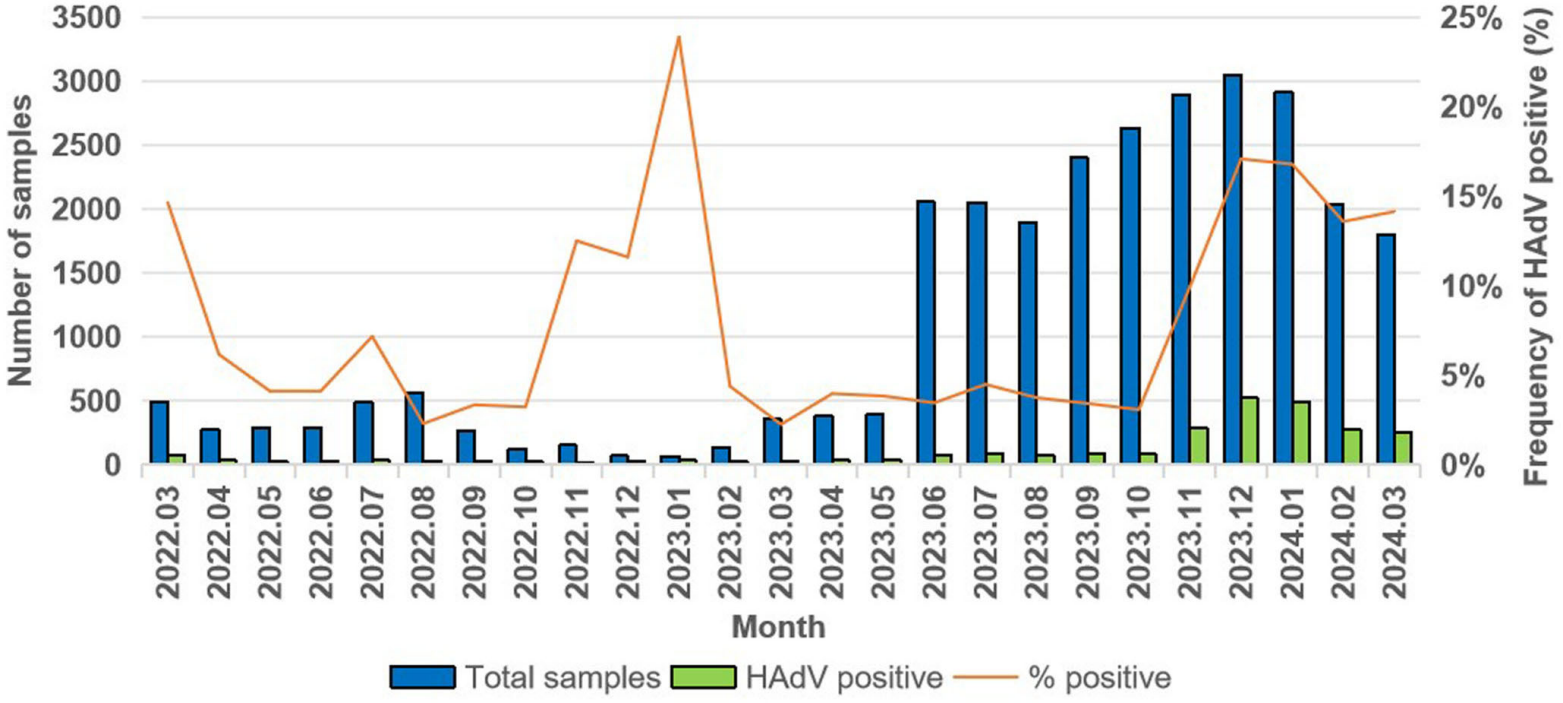
Monthly distribution of HAdV infection in children with ARIs in Tianjin from March 2022 to March 2024.

A total of 28,060 children with ARIs were enrolled in the study, and 2,484 cases (8.9%) were identified as positive for HAdV. Among these, 1,435 (57.8%) were male and 1,049 (42.2%) were female. The detection rate of HAdV was higher in males (9.2%, 1435/15584) than in females (8.4%, 1049/12476), and the difference was significant (*χ²* =5.496, *P* = 0.019). All cases were categorized into four age groups: infants (<1 year), toddlers (1–<3 years), preschoolers (3–<6 years), and school-aged children (6–<18 years). Among all age groups, the HAdV detection rate was highest in the 6–18 years age group (10.4%), and the difference was statistically significant (*χ²* =97.022, *P* < 0.001). HAdV infections were detected throughout the year, and a significant difference was observed in the HAdV detection rate across seasons (*χ²* =834.146, *P* < 0.001), with the highest detection rate of HAdV in winter (15.9%, 1315/8266). To investigate the temporal trends in HAdV prevalence, we analyzed the monthly distribution of positive samples. Notably, significant peaks in detection rates were observed during specific months: January 2023 (23.9%, 16/67), December 2023 (17.1%, 520/3046), and January 2024 (16.8%, 489/2914). In contrast, strikingly low HAdV positivity was detected in August 2022 (2.3%, 13/566) and March 2023 (2.3%, 8/355). The clinical and epidemiological data are shown in **Table 1** and **Figure 2**.

**Table 1:** HAdV-positive in children of different ages and gender with ARIs.

Variable	Total ARIs (N (%))	HAdV-positive (N (%))	*χ^2^*	*P*
Sex			5.496	**0.019**
Male	15584 (55.5)	1435 (9.2)		
Female	12476 (44.5)	1049 (8.4)		
Age group			97.022	**< 0.001**
Infant (<1 Year)	7323 (26.1)	458 (6.3)		
Toddler (1 Year to <3 Year)	5332 (19.0)	456 (8.6)		
Preschool (3 Year to <6 Year)	7113 (25.3)	708 (10.0)		
School (6 Year to <18 Year)	8292 (29.6)	862 (10.4)		
Season			834.146	**< 0.001**
Spring (Mar-May)	3981 (14.2)	393 (9.9)		
Summer (Jun-Aug)	7347 (26.2)	294 (4.0)		
Autumn (Sep-Nov)	8466 (30.2)	482 (5.7)		
Winter (Dec-Feb)	8266 (29.5)	1315 (15.9)		
Total	28060 (100.00)	2484 (8.9)		

Values showing statistically significant differences are indicated in bold (P < 0.05).

### 3.3 Distribution characteristics of HAdV genotypes

**Figure 3:**
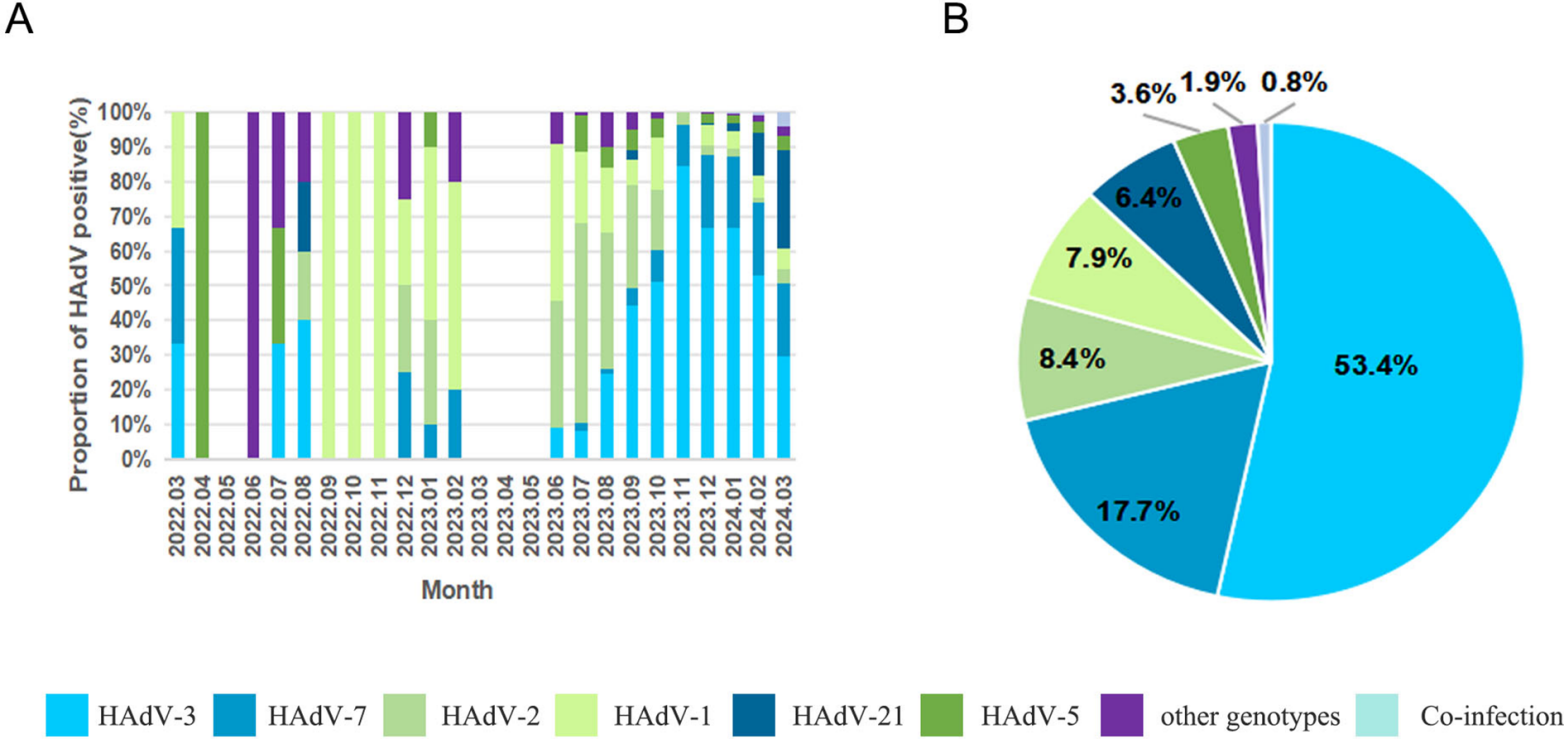
The distributions of human adenovirus types detected in children with acute respiratory infections. **(A)** HAdV genotype distribution of ARI in patients by month; **(B)** HAdV genotype distribution of ARI inpatients.

A total of 1914 specimens were successfully genotyped, revealing 13 distinct HAdV types spanning species B, C, D, and E (**Figure 3A**). The epidemiological profile demonstrated HAdV-3 as the predominant type (53.4%, 1022/1914), followed in descending order by HAdV-7 (17.7%, 338/1914), HAdV-2 (8.4%, 160/1914), HAdV-1 (7.9%, 152/1914), HAdV-21 (6.4%, 122/1914), and HAdV-5 (3.6%, 68/1914). Sporadic cases (<1% prevalence) included HAdV-4 (17/1914), HAdV-6 (10/1914), HAdV-57 (3/1914), HAdV-55 (2/1914), HAdV-108 (2/1914), HAdV-8 (1/1914), and HAdV-31 (1/1914). Notably, 16 co-infection cases were characterized, with HAdV-1/HAdV-2 (*n*=7) was the most frequent, followed by HAdV-1/HAdV-3 (n=2), HAdV-1/HAdV-5 (*n*=2), HAdV-2/HAdV-5 (*n*=2), with single occurrences of HAdV-3/HAdV-21, HAdV-3/HAdV-7, and HAdV-5/HAdV-7. Temporal analysis revealed that samples genotyped prior to August 2023 exhibited a higher prevalence of the HAdV-C species, whereas those genotyped after August 2023 showed a dominance of the HAdV-B species (**Figure 3B**).

### 3.4 Demographic and clinical characteristics of different HAdV genotypes

#### 3.4.1 HAdV genotypes according to age

**Figure 4:**
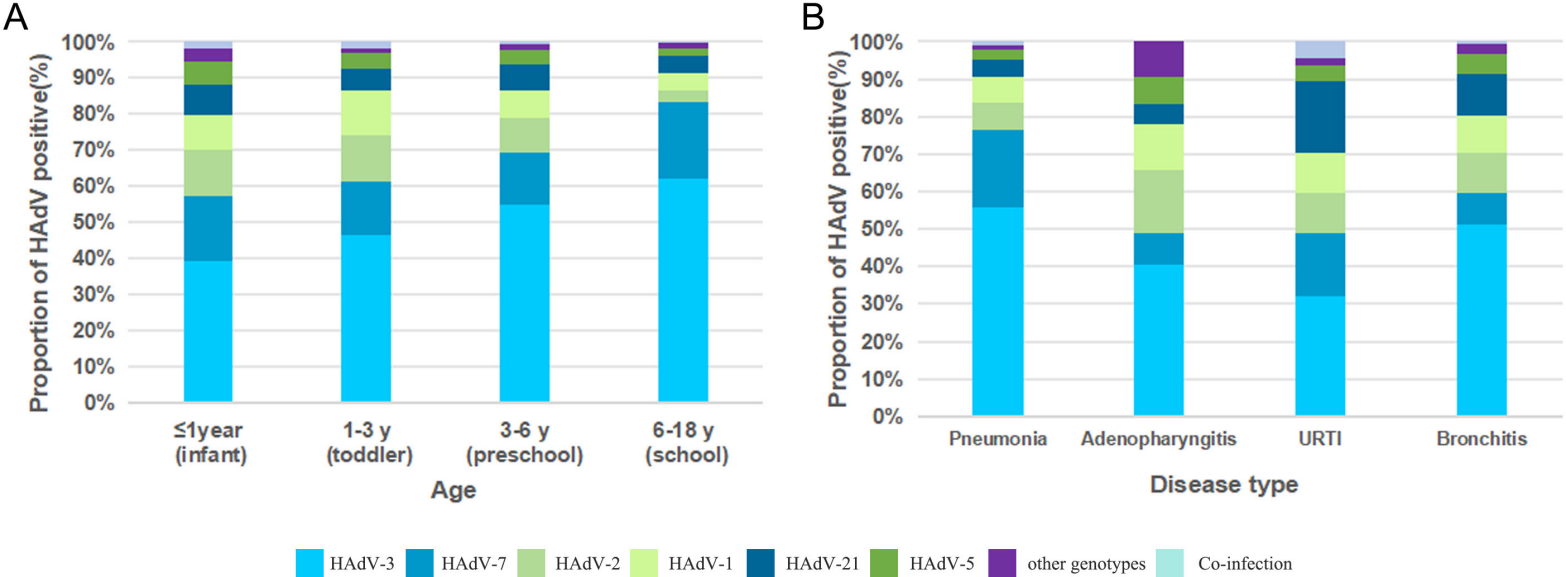
**(A)** HAdV genotype distribution of ARI inpatients by age; **(B)** HAdV genotype distribution of ARI inpatients by disease type.

To investigate age-specific epidemiological patterns of HAdV infection in children, patients were stratified into four distinct age categories: infants (<1 year of age), toddlers (1**–**3 years), preschoolers (3**–**6 years), and school-aged children (6**–**18 years). Significant differences in HAdV type distribution were observed across age groups (*χ²*=111.479, *P* < 0.001). The HAdV-3/HAdV-7 dominance pattern persisted across all age strata, particularly accentuated in school-aged children, where HAdV-3 accounted for 62.2% of cases, followed by HAdV-7 at 21.2%. Moreover, distinct age-specific prevalence peaks were observed. HAdV-2, -5, and -21 showed maximal detection during infancy (13.7%, 6.9%, 9.0%), while HAdV-1 demonstrated its highest infection rate in toddlers (12.2%) (**Figure 4A**).

#### 3.4.2 HAdV genotypes according to the disease type

Significant differences in HAdV-type distribution were observed among various clinical diagnoses (*χ²* =104.385, *P* < 0.001). Analysis of 1,914 confirmed cases revealed pneumonia as the predominant diagnosis (72.2%, 1,382/1,914), followed by bronchitis (20.3%, 389/1,914). In contrast, adenopharyngitis (5.0%, 96/1,914) and URTIs (2.5%, 47/1,914) represented relatively smaller proportions of the total cases. Notably, HAdV-3 maintained predominance across all disease entities, particularly in pneumonia (55.6%, 769/1382) and bronchitis (51.2%, 199/389). Diagnosis-specific secondary genotypes emerged: HAdV-7 constituted 20.9% (289/1382) of pneumonia cases, while HAdV-2 accounted for 16.7% (16/96) of adenopharyngitis cases. Notably, HAdV-21 demonstrated dual clinical relevance, representing 11.1% (43/389) of bronchitis cases and 19.1% (9/47) of URTIs (**Figure 4B**).

#### 3.4.3 Clinical characteristics of different HAdV types infections in children with pneumonia

Focusing on the predominant HAdV-3 (*n*=769) and HAdV-7 (*n*=289) pneumonia cases, we conducted comparative analyses with other HAdV types of pneumonia (*n*=324). No sex-based differences were observed across groups (*χ²* =1.469, *P* =0.480). Age stratification revealed a significant difference (*χ²* =106.821, *P* < 0.001), with school-aged children predominating in both HAdV-3 (62.4%, 337/540) and HAdV-7 (23.7%, 128/540). The clinical symptoms comprised fever (94.1%), cough (98.7%), and hyperpyrexia (74.0%), with frequent erythra and lymphadenopathy. Further analysis found that patients infected with HAdV-7 exhibited marked clinical severity, showing longer Duration of hospitalization, prolonged fever duration, and higher incidence of high fever than other groups (*χ²* =18.93, *P* < 0.001; *χ²* =24.369, *P* < 0.001; *χ²* =28.165, *P* < 0.001). These findings collectively indicated that HAdV-7 infections were more likely to develop severe pneumonia (*χ²* =42.434, *P* < 0.001). In addition, transcutaneous oxygen saturation levels in this group were significantly lower compared to other groups (*χ²* =11.585, *P* =0.003). Gastrointestinal symptoms were also observed in a subset of patients, including vomiting, diarrhea, abdominal pain. Wheezing demonstrated a higher prevalence in children infected with other types of HAdV (*χ²* =8.5, *P* =0.014).

Within the HAdV-7 cohort, a higher prevalence of underlying conditions was observed among patients, such as anemia (n=13), immunodeficiency (n=3), congenital heart disease (n=9), epilepsy (n=2), fatty liver (n=9), developmental delays (n=1), and growth retardation (n=1). Furthermore, intrapulmonary complications occurred more frequently in HAdV-7 infection cases compared to other types HAdV. Specifically, the incidence of pulmonary atelectasis (*χ²* =8.797, *P* =0.012), pleurisy (*χ²* =11.344, *P* =0.003), pleural effusion (*χ²* =19.629, *P* < 0.001), and plastic bronchitis (*χ²* =27.587, *P* < 0.001) was significantly higher in this group than other groups. Extrapulmonary complications also showed a higher incidence in cases of HAdV-7 infection, particularly abnormal coagulation function, liver injury, and gastrointestinal dysfunction. Statistical analysis found that there were significant differences among the three groups in terms of the incidence of myocardial injury (*χ²* =13.166, *P <*0.001), toxic encephalopathy, or encephalitis (*χ²* =10.236, *P* =0.004). Importantly, children infected with HAdV-7 have a higher probability of receiving immunoglobulin therapies and fiberoptic bronchoscopy procedures compared to other groups (*χ²* =24.333, *P* < 0.001; *χ²*=93.644, *P* < 0.001) (**Table 2**).

**Table 2:** Clinical manifestations and outcomes among children hospitalized with adenovirus pneumonia.

Characteristics	Group				*χ²*/Z	*P*
	HAdVs (n=1382)	HAdV-3 (n=769)	HAdV-7 (n=289)	Others (n=324)		
Gender					1.469	0.48
Male	796 (57.6)	437 (56.8)	163 (56.4)	196 (60.5)		
Female	586 (42.4)	332 (43.2)	126 (43.6)	128 (39.5)		
Age (year)					106.821	**<0.001**
≤1y (infant)	258 (18.7)	111 (14.4)	57 (19.7)	90 (27.8)		
1–3 y (toddler)	220 (15.9)	112 (14.6)	43 (14.9)	65 (20.1)		
3–6 y (preschool)	364 (26.3)	209 (27.2)	61 (21.1)	94 (29.0)		
6–18 y (school)	540 (39.1)	337 (43.8)	128 (44.3)	75 (23.1)		
Clinical manifestation						
Duration of hospitalization(d) [M (P25, P75)]	5 (4,7)	5 (4,7)	6 (4.5,7)	5 (4,6)	18.93	**<0.001**
Fever [n (%)]	1300 (94.1)	729 (94.8)	279 (96.5)	292 (90.1)	12.931	**0.002**
Hyperpyrexia (≥ 39°C) [n (%)]	1022 (74.0)	593 (77.1)	226 (78.2)	203 (62.7)	28.165	**<0.001**
Highest temperature (°C) [M (P25, P75)]	39.4 (38.9,40)	39.5 (39,40)	39.4 (39,40)	39 (38.5,39.6)	42.91	**<0.001**
Duration of fever(d) [M (P25, P75)]	4 (3,7)	5 (3,7)	5 (3,7)	3 (2,6)	24.369	**<0.001**
Breathe (times/min) [M (P25, P75)]	22 (24,27)	21 (24,26)	21 (24,27)	22 (24,28)	2.077	0.126
SpO2(%)	98 (97,98)	98 (97,98)	98 (96,98)	98 (96.25,98)	11.585	**0.003**
Cough [n (%)]	1364 (98.7)	760 (98.8)	286 (99)	318 (98.1)	1.059	0.589
Wheezing [n (%)]	109 (7.9)	48 (6.2)	24 (8.3)	37 (11.4)	8.5	**0.014**
Conjunctivitis [n (%)]	39 (2.8)	24 (3.1)	3 (1.0)	12 (3.7)	4.523	0.104
Vomiting [n (%)]	241 (17.4)	128 (16.6)	58 (20.1)	55 (17.0)	1.774	0.412
Chest pain [n (%)]	20 (1.4)	7 (0.9)	8 (2.8)	5 (1.5)	4.913	0.069
Stomachache [n (%)]	115 (8.3)	74 (9.6)	28 (9.7)	13 (4.0)	10.301	**0.006**
Diarrhea [n (%)]	60 (4.3)	25 (3.3)	15 (5.2)	20 (6.2)	5.32	0.07
Erythra [n (%)]	90 (6.5)	54 (7.0)	15 (5.2)	21 (6.5)	1.158	0.573
Headache [n (%)]	37 (2.7)	25 (3.3)	7 (2.4)	5 (1.5)	2.643	0.264
Lymphadenopathy [n (%)]	70 (5.1)	50 (6.5)	5 (1.7)	15 (4.6)	10.114	**0.006**
Comorbid disease [n (%)]^a^	122 (8.8)	56 (7.3)	38 (13.1)	28 (8.6)	9.001	**0.011**
Pulmonary complications						
Emphysema [n (%)]	14 (1.0)	7 (0.9)	4 (1.4)	3 (0.9)	0.687	0.714
Pulmonary atelectasis [n (%)]	112 (8.1)	65 (8.5)	32 (11.1)	15 (4.6)	8.797	**0.012**
Pleurisy [n (%)]	373 (27.0)	204 (26.5)	98 (33.9)	71 (21.9)	11.344	**0.003**
Pleural effusion [n (%)]	74 (5.4)	35 (4.6)	30 (10.4)	9 (2.8)	19.629	**<0.001**
Respiratory failure [n (%)]	5 (0.4)	3 (0.4)	2 (0.7)	0	1.886	0.321
Necrotizing pneumonia [n (%)]	6 (0.4)	4 (0.5)	1 (0.3)	1 (0.3)	0.263	1
Plastic bronchitis [n (%)]	63 (4.6)	32 (4.2)	28 (9.7)	3 (0.9)	27.587	**<0.001**
Extrapulmonary complications						
Liver injury [n (%)]	65 (4.7)	36 (4.7)	19 (6.6)	9 (2.8)	4.148	0.126
Myocardial injury [n (%)]	19 (1.4)	7 (0.9)	11 (3.8)	1 (0.3)	13.166	**0.001**
Abnormal coagulation function [n (%)]	204 (14.8)	128 (16.6)	46 (15.9)	30 (9.3)	10.271	**0.006**
Gastrointestinal dysfunction [n (%)]	94 (6.8)	52 (6.8)	26 (9.0)	16 (4.9)	3.973	0.137
Toxic encephalopathy or encephalitis	21 (1.5)	7 (0.9)	11 (3.8)	3 (0.9)	10.236	**0.004**
Urinary system injury [n (%)]	17 (1.2)	11 (1.4)	2 (0.7)	4 (1.2)	0.771	0.755
Pyemia [n (%)]	17 (1.2)	13 (1.7)	3 (1.0)	1 (0.3)	3.52	0.156
Severe pneumonia [n (%)]	364 (26.3)	197 (25.6)	114 (39.4)	53 (16.4)	42.434	**<0.001**
Treatment						
Immunoglobulin therapy [n (%)]	132 (9.6)	70 (9.1)	47 (16.3)	15 (4.6)	24.333	**<0.001**
Bronchoscope [n (%)]	525 (38.0)	315 (41.0)	155 (53.6)	55 (17.0)	93.644	**<0.001**
Endotracheal intubation [n (%)]	9 (0.7)	6 (0.8)	3 (1.0)	0	2.99	0.23

Values showing statistically significant differences are indicated in bold (*P* < 0.05)

a Including anemia, immune deficiency, congenital heart disease, malnutrition, epilepsy, fatty liver, developmental delay, and underdevelopment.

### 3.5 Phylogenetic analysis

**Figure 5:**
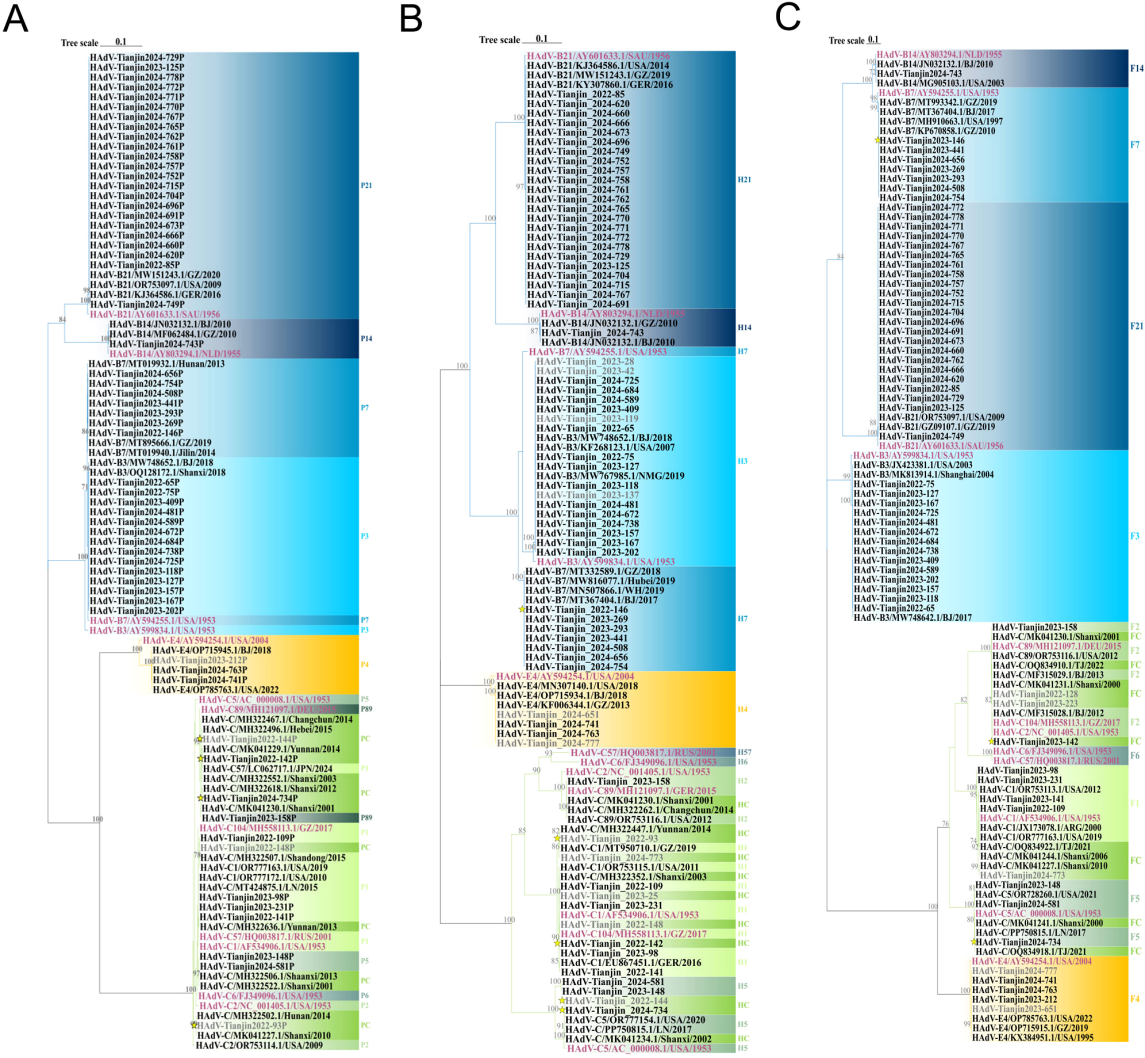
Phylogenetic tree constructed on the basis of the nucleotide sequences of the Penton base **(A)**, Hexon **(B)** and Fiber **(C)** gene sequences of the isolation. Additional reference sequences were retrieved from GenBank to provide context and reference. The original strains are marked in purple, strains that failed to successfully amplify three gene sequences simultaneously are marked in gray, strains with inconsistent gene sequence typing results are represented. by yellow stars. The phylogenetic tree is constructed using ML method, and the values at the branch nodes represent the Bootstrap support for 1000 repetitions (only displaying values ≥ 70%).

To further analyze the HAdV genotype, the Hexon, Penton base, and Fiber genes of 70 HAdV-positive samples were amplified using conventional PCR in this study. Finally, the three genes of 56 samples were successfully sequenced simultaneously. Comparative analysis of sequence homology between established HAdV genotypes and their prototype strains revealed consistently high nucleotide and amino acid conservation across all examined genetic lineages (**Table 3**). Phylogenetic analysis of HAdV Hexon, Penton base, and Fiber genes demonstrated the genotyping results of 54 samples were consistent, wherein the identified HAdV types were distributed as follows: HAdV-3 (25.9%, 14/54), HAdV-7 (13.0%, 7/54), HAdV-21 (42.6%, 23/54), HAdV-1 (7.4%, 4/54), HAdV-4 (3.7%, 2/54), HAdV-5 (3.7%, 2/54), HAdV-89 (1.9%, 1/54), and HAdV-14 (1.9%, 1/54). However, the Penton gene sequences from the remaining two cases (TJ2024-734, H5F5; TJ2023-142, H1F2) could not be definitively typed (**Figure 5**).

**Table 3:** The nucleotide and amino acid identified of Penton, Hexon and Fiber.

Species	Genotypes	Percent identity of nucleotide (nt%)			Percent identity of amino acid (nt%)		
		Penton	Hexon	Fiber	Penton	Hexon	Fiber
HAdV-B	HAdV-3	98.1-98.2	98.6-98.7	98.3-98.4	98.7-98.9	98.6-98.7	96.3-96.6
	HAdV-7	99.1	95.7	99.3	99.1	96.8	98.5
	HAdV-14	99.8	99.8	99.9	99.0	99.3	98.8
	HAdV-21	99.3-99.4	98.9-99.1	99.3-99.4	93.7-94.2	97.2-98.0	98.8-99.1
HAdV-C	HAdV-1	99.6-99.8	99.8-99.9	98.9-99.8	99.7-99.8	99.5-99.8	98.3-99.5
	HAdV-89	99.4	99.9	99.9	99.7	99.6	100
	HAdV-5	98.4-98.5	95.5	99.5-99.6	98.2	98.5	99.5
HAdV-E	HAdV-4	96.4	97.6	98	96.1	97.5	97.9

#### 3.5.1 Phylogenetic tree of the Penton gene

**Figure 6:**
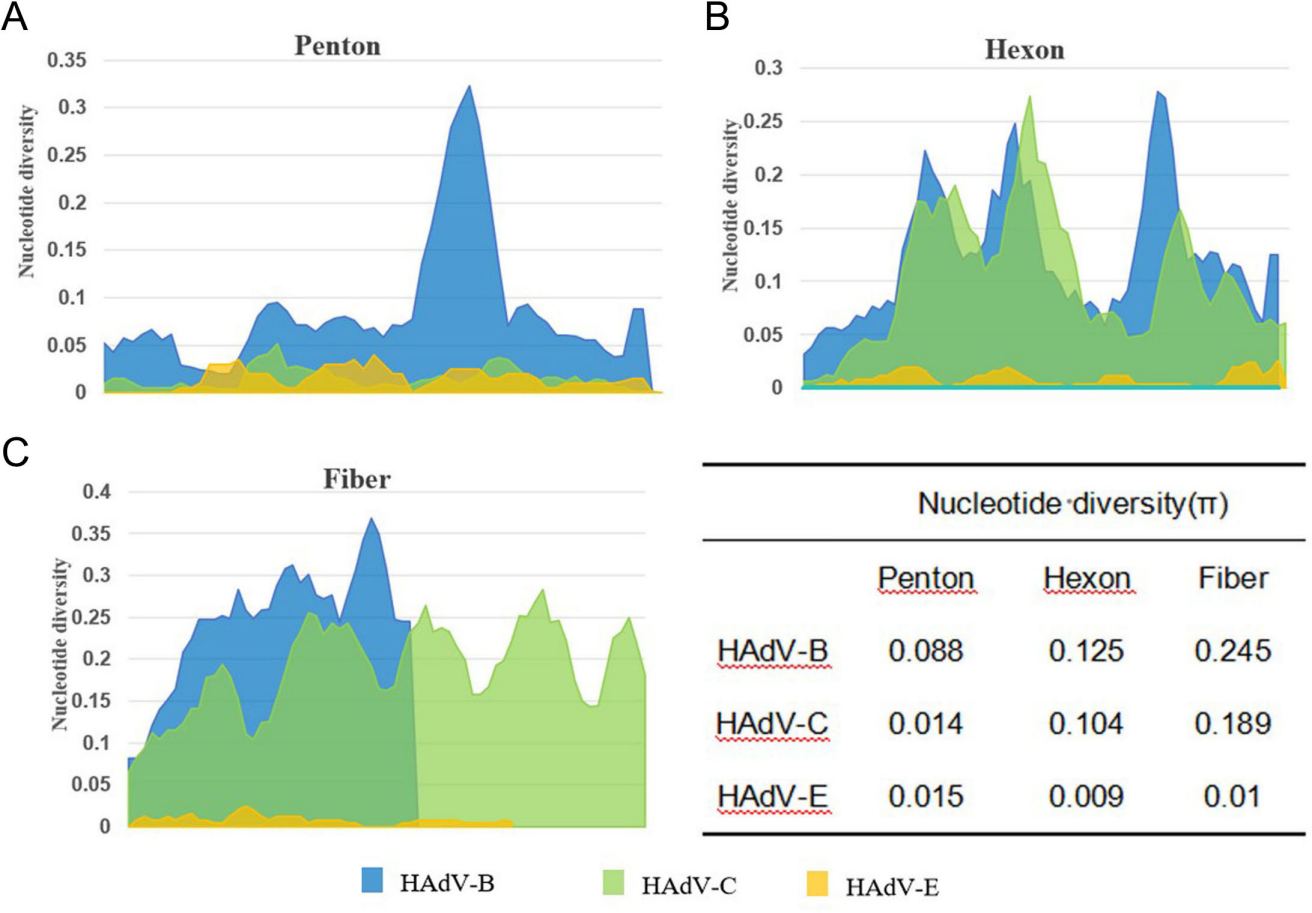
Nucleotide diversity plots showing the average number of nucleotide differences per site along each gene for HAdV-B, C and E, calculated for Penton base **(A)**, Hexon **(B)**, and Fiber **(C)** prototype sequences.

In the Penton-based phylogenetic tree, the 13 HAdV-3 sequences obtained in this study, except for TJ2024-481, were completely identical and clustered with BJ2018 (MW748652.1) and SX2018 (OQ128172.1), with an average genetic distance of 0.0025 within the cluster. The 7 HAdV-7 sequences obtained in this study were entirely identical and clustered tightly with GZ2019 (MT895666.1) and JL2014 (MT019940.1), showing an average intra-cluster genetic distance of 0.0024. Interestingly, the Penton gene of the HAdV-3 isolate exhibited relatively high similarity to its prototype strain, reaching 98.1%-98.2%. However, it did not form a cluster with the prototype. Further nucleotide concordance analysis demonstrated that the HAdV-3 Penton sequences in this study showed even higher concordance with both the HAdV-7 prototype (99.2%-99.3%) and studied sequences (99.5%). The sole HAdV-14 clinical isolate clustered robustly with BJ2010 (JN032132.1) and GZ2010 (MF062484.1), presenting an average intra-cluster genetic distance of 0.0020. Additionally, all 22 HAdV-21 sequences, except TJ2024-749, were entirely identical and grouped into a clade with GZ2020 (MW151243.1) and USA2009 (OR753097.1), with an average intra-cluster genetic distance of 0.0017. For HAdV-4, the three Penton sequences obtained in this study were completely identical and clustered tightly with BJ2018 (OP715945.1) and USA2022 (OP785763.1), these sequences showed only 96.4% nucleotide identity with their prototype strain, and the average intra-cluster genetic distance was calculated as 0.0242. Phylogenetic analysis further revealed that HAdV-C strains could not be fully differentiated based on Penton gene sequences alone, with an average intra-cluster genetic distance of 0.0146 (**Figure 5A**). A comparative analysis of nucleotide polymorphisms indicated that the Penton gene of HAdV-C exhibited a lower nucleotide diversity (1.4%) compared to Hexon (10.4%) and Fiber (18.9%). Furthermore, the Penton gene nucleotide diversity of HAdV-C species was lower than that of HAdV-B (8.8%) and HAdV-E (1.5%) (**Figure 6**).

#### 3.5.2 Phylogenetic tree of the Hexon gene

**Figure 7:**
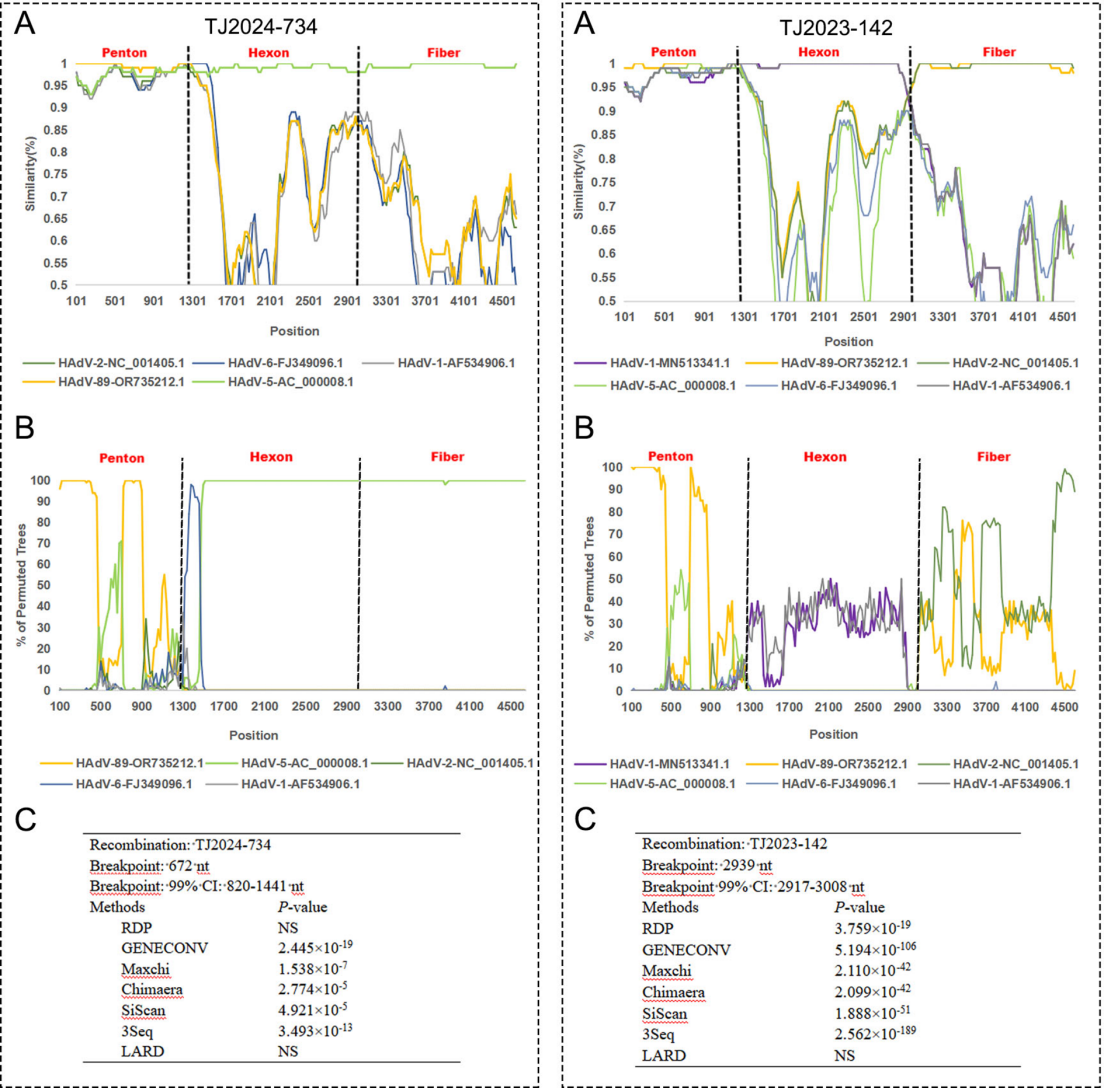
Recombination analysis of TJ2024–742 and TJ2023–142 with other HAdV-C species using SimPlot **(A)**, BootScan **(B)** and RDP **(C)**. The analysis supports TJ2024–742 as a recombinant strain.

Phylogenetic analysis of the Hexon gene indicated that three species of HAdV, including 11 different types, were identified throughout the study period (**Figure 7**). In this study, the HAdV-3 isolates clustered with BJ2018 (MW748652.1) and NMG2019 (MW767985.1), exhibiting an average genetic distance of 0.0022 within the group. The six HAdV-7 sequences were completely identical except for TJ2024-656. However, they did not cluster with the prototype strain, displaying a nucleotide sequence identity of only 95.7%, resulting in an average genetic distance of 0.0115 within the group. The HAdV-14 strain obtained clustered with GZ2010 (MF062484.1) and BJ2010 (JN032132.1), showing an average genetic distance of 0.0009. Additionally, the 23 HAdV-21 isolates exhibited a nucleotide similarity ranging from 99.9% to 100.0%, clustering with GZ2019 (MW151243.1) and presenting an average genetic distance of 0.0009 within the group. The 3 HAdV-4 sequences, with the exception of TJ2024-651, were identical and clustered with BJ2018 (OP715934.1) and GZ2013 (KF006344.1), with an average genetic distance of 0.0152 within the group. Furthermore, both HAdV-1 and 2 were found to be capable of being divided into two distinct branches. Interestingly, the HAdV-1 sequences obtained in this study primarily clustered with the prototype strains to form cluster 1. However, two samples, TJ2022–93 and TJ2024-773, grouped with YN2014 (MH322447.1) and GZ2019 (MT950710.1) to form cluster 2. The average genetic distances within the groups of cluster 1 and cluster 2 were 0.0043 and 0.0015, respectively, and the average genetic distances between groups was 0.0065. The HAdV-2 prototype strains formed a separate cluster, while the TJ2023–158 from our study and HAdV-89 prototype strains, along with SX2001 (MK041230.1), CC2014 (MH322262.1), and USA2012 (OR753116.1), were grouped into another cluster. The average genetic distance between this cluster and the HAdV-2 prototype strain was 0.0074. The 4 HAdV-5 sequences obtained were divided into two clusters. In the first cluster, the sequences of TJ2024–581 and TJ2023–148 were identical, showing 95.5% nucleotide sequence identity to the prototype strain. In the second cluster, TJ2024–734 and TJ2022–144 were identical and clustered with SX2002 (MK041234.1) and LN2017 (PP750815.1), displaying 99.2% nucleotide sequence identity with the prototype strain. The average genetic distance within this cluster was 0.0259 (**Figure 5B**).

#### 3.5.3 Phylogenetic tree of the Fiber gene

In the phylogenetic analysis of the Fiber gene, a clear separation of genotypes was well-supported. Among the 14 HAdV-3 sequences obtained, a discrepancy was observed only at nucleotide position 214. These sequences clustered with SH2004 (MK813914.1) and BJ2017 (MW748642.1), with an average genetic distance of 0.0026 within the group. The 7 HAdV-7 sequences exhibited complete congruence and clustered with GZ2010 (KP670858.1) and BJ2017 (MT367404.1), showing an average genetic distance of 0.0020. The HAdV-14 sequence shared a cluster with BJ2010 (JN032132.1), displaying an average genetic distance of 0.0010. Excluding TJ2024-749, the 22 sequences of HAdV-21 were entirely consistent and clustered with GZ2019 (GZ09107.1) with an average genetic distance of 0.0006. The 5 HAdV-4 sequences were also found to be completely consistent, clustering alongside GZ2019 (OP715915.1), with an average genetic distance of 0.0078. Interestingly, HAdV-1 was categorized into two clusters, with the 4 HAdV-1 sequences showing a consistency of 98.1% to 99.9%, resulting in an average genetic distance of 0.0109 within the cluster. Additionally, TJ2023–158 clustered with the HAdV-89 prototype strain SX2001 (MK041230.1), while TJ2022-128, TJ2023-142, and TJ2023–223 clustered with the HAdV-2 prototype strains BJ2012 (MF315028.1) and GZ2017 (MH558113.1). The nucleotide identity among these four sequences ranged from 99.3% to 99.8%, with an average genetic distance of 0.0031 within the cluster. The three HAdV-5 sequences obtained were 99.3%-99.9% concordant and clustered with LN2017 (PP750815.1), TJ2021 (OQ834919.1), with an average genetic distance of 0.0042 within the cluster (**Figure 5C**).

### 3.6 Recombination analysis

For strains TJ2024–734 and TJ2023-142, multiple algorithms within RDP5 (≥5 algorithms) consistently and highly significantly (p << 0.01) detected identical recombination breakpoint positions and identical parental combinations (**Figure 7**).

SimPlot and BootScan analyses for TJ2024–734 revealed clear evidence of recombination within its concatenated Penton-Hexon-Fiber sequence relative to reference strains OR735212.1 and AC_000008.1. Upstream of approximately nt 1000, TJ2024–734 exhibited strong bootstrap support (>75%) with reference strain OR735212.1. Downstream of approximately nt 1300, high bootstrap support (>90%) shifted to reference strain AC_000008.1. This sharp transition around nt 1000–1300 indicates a recombination breakpoint, consistent with the RDP-predicted breakpoint location (nt 820-1141). The SimPlot profile clearly showed distinct peaks of sequence similarity to the different reference strains across the genomic regions, strongly indicating that TJ2024–734 is a recombinant strain.

For TJ2023-142, SimPlot and BootScan analyses revealed distinct phylogenetic patterns across different genomic regions: the Penton region showed strong bootstrap support (>75%) for reference strain OR735212.1. The Hexon region exhibited consistently low bootstrap support (<50%) across all reference strains, although MN513341.1 displayed slightly higher affinity (~50%). The Fiber region showed moderate bootstrap support (40-75%) for OR735212.1. Despite the reduced bootstrap support in the Hexon region, several lines of evidence strongly suggest TJ2023–142 is a recombinant strain (1): RDP5 consistently detected a highly significant recombination breakpoint (p < 0.01) at nt 2917-3008 (Hexon-Fiber junction) (2); SimPlot analysis showed nearly 100% sequence identity between the TJ2023–142 Hexon region and MN513341.1; and (3) the Penton and Fiber regions maintained high sequence similarity (Bootstrap >75%) to OR735212.1.

### 3.7 Amino acid mutation analysis

**Figure 8:**
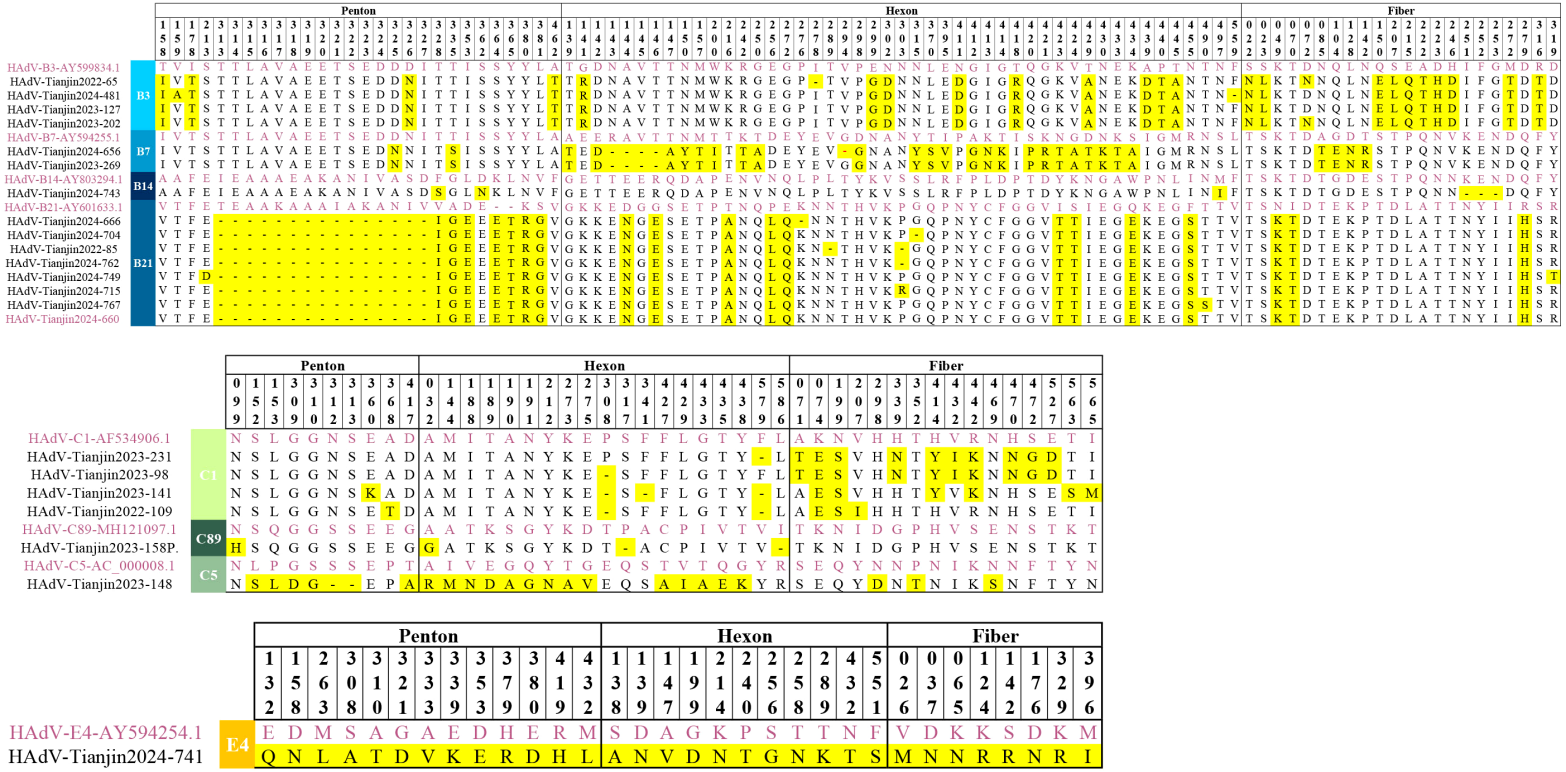
Amino acid alignment view of the Hexon, Penton and Fiber proteins from the selected HAdV sequences representing different genotypes. The original strains are marked in purple, the mutated amino acids are highlighted in yellow.

Using prototype strains as references, we systematically characterized amino acid polymorphisms across the Penton base, Hexon, and Fiber genes of HAdV (**Figure 8**). Key findings revealed that Hexon protein exhibited the highest variability with 85 polymorphic sites. Penton protein showed intermediate diversity (55 polymorphic sites). Fiber protein demonstrated the lowest variation (46 polymorphic sites). Notably, amino acid substitutions predominated over deletions in all gene products except for the HAdV-21 Penton protein, where deletion events occurred more frequently. In addition, HAdV-21 and HAdV-7 displayed a higher polymorphism rates (41 and 34 variants, respectively). But, HAdV-89 showed remarkable sequence conservation with only 4 variants detected.

#### 3.7.1 Amino acid mutation analysis of Penton

The Penton protein contains the Arg-Gly-Asp (RGD) sequence, which interacts with cellular integrins and thereby mediates viral endocytosis. When compared to the prototype strain, HAdV- B species exhibited 32 polymorphic sites, HAdV-C species had 10 polymorphic sites, and HAdV-E species displayed 13 polymorphic sites. Importantly, all 23 cases of HAdV-21 obtained in this study had 15 consecutive amino acid deletions at positions 313–327 within the RGD loop and two amino acid insertions downstream of the RGD motif. Compared with the prototype, it maintains 99.3-99.4% nucleotide conservation while reducing amino acid identity to 93.7-94.2%.

#### 3.7.2 Amino acid mutation analysis of Hexon

The Hexon protein serves as the major antigenic component of HAdV and forms the basis for typing in neutralization assays. In comparison to the prototypic strains, HAdV-B species exhibited 54 polymorphic sites, HAdV-C species had 20 polymorphic sites, and HAdV-E species displayed 11 polymorphic sites. Notably, HAdV-7 possessed the highest number of polymorphic sites with 28 sites, comprising 5 deletions and 23 substitutions. The amino acid identity of HAdV-7 is only 96.8% compared to the prototype strain.

#### 3.7.3 Amino acid mutation analysis of Fiber

The Fiber protein serves as the foundation for typing in hemagglutination inhibition assays and contains type-specific antigenic epitopes that determine viral tropism. Variants in this region may result in different histophilicity. In comparison to the prototypic strains, HAdV-B species exhibited 22 polymorphic sites, HAdV-C species had 16 polymorphic sites, and HAdV-E species displayed 8 polymorphic sites. Notably, no amino acid deletions were found in any of the strains except for HAdV-14, which had three consecutive amino acid deletions at positions 251-253.

## 4 Discussion

HAdV infection represents a significant etiology of ARIs in children, accounting for approximately 4%-10% of global pediatric ARIs cases (Yu et al., 2021; Zhu et al., 2021). Our surveillance in Chinese cities revealed substantial spatiotemporal variation in HAdV detection. Among hospitalized pediatric ARIs patients in Tianjin, the HAdV positive rate was 8.9%, significantly higher than Beijing (2.89%, 2015-2021) but lower than Hangzhou (26.77%, 2020-2021) (Huang et al., 2023; Wang et al., 2024).Longitudinal analysis showed cyclical fluctuations in Tianjin, peaking at 12.22% in 2019, aligning with the national trend (Liu et al., 2022; Wang et al., 2024). The implementation of NPI measures from 2020 to 2022 substantially suppressed HAdV transmission (declining to 7.82% in 2020, 172/2199). However, a resurgence was observed in 2021 as social activities resumed (9.69%, 897/9253) (**Supplementary Figure S1**). Parallel trends were documented in other urban centers (Wang et al., 2024). During the NPI period, the HAdV positivity rate in Tianjin was 8.78% (1,270/1,4471). Following the lifting of the NPI, there was a significant increase in both the number of ARIs patients and the absolute number of HAdV cases. However, the HAdV positivity rate remained stable at 9.12% (2,283/25,041), in contrast to the upward trend observed in Beijing and Hebei (Zhao et al., 2024; Han et al., 2025). This ‘asynchronous change’ highlights the complexity of transmission dynamics in the context of co-circulation of multiple pathogens (such as RSV) (Lyu Y and Zhang, 2024).

HAdV positivity exhibited significant differences by age, gender, and season in Tianjin. Infections peaked in autumn and winter (December-January), consistent with seasonal patterns in northern temperate regions (Huang et al., 2021; Yu et al., 2021), with HAdV-3 predominating (53.4%). Contrary to previous studies reporting higher prevalence in children under 5 (Barnadas et al., 2018; Zhao et al., 2020; Huang et al., 2021; Pscheidt et al., 2021), our study found the lowest rate in 0–1 year-olds (6.25%) and the highest in 6–18 year-olds (10.4%). This shift may relate to reduced intra-family transmission during NPIs (decreasing infant exposure) and increased transmission within schools post-reopening (Huang et al., 2023; Yan et al., 2024). Gender difference analysis showed that there were more hospitalized male ARIs patients in this study, which may be related to biological factors (boys with relatively narrower airway diameters are more prone to severe lung infections (Pagtakhan et al., 1984)) and behavioral factors (girls have higher compliance with mask wearing during NPI (Huang et al., 2023)). The HAdV positivity rate was significantly higher in males (9.2% *vs*. 8.4%; *χ*² = 5.496, *P* = 0.019), consistent with some studies (Pscheidt et al., 2021; Huang et al., 2023) but not others (Wang et al., 2021; Liu et al., 2022). The reasons for gender differences warrant further investigation. Clinically, male HAdV patients may require heightened vigilance for severe symptoms and pneumonia progression. Molecular epidemiology showed a shift in predominant types: HAdV-C dominated during NPIs (typically milder), rapidly replaced by HAdV-B (78.2%) post-NPIs, often associated with more severe illness (Huang et al., 2023; Wang et al., 2024). This indicates NPIs not only affect transmission but also viral type composition and community disease severity, underscoring the need for long-term subtype surveillance and early warning.

HAdV-C infections were predominantly observed in children under 3 years old (206/395, 52.2%), whereas HAdV-B3/B7 infections were more prevalent among school-aged children. In addition to the age-specific distribution, adenovirus serotypes significantly influenced disease phenotypes and severity. HAdV-3 predominated across all disease categories, especially pneumonia and bronchitis. Previous study revealed that HAdV-C frequently resides latently in adenoid tissues (Alkhalaf et al., 2013); thus, it is the second most common serotype observed in tonsillitis patient samples. Although HAdV-3 displayed higher overall prevalence, children infected with HAdV-7 more commonly progressed to severe symptoms, which may be attributed to its replication advantages: *in vitro* experiments demonstrated that HAdV-7 exhibits a higher viral load compared to HAdV-3 and induces stronger cytokine responses. Additionally, infections with HAdV-7 were more frequently associated with complications such as myocardial injury and toxic encephalopathy or encephalitis. Patients infected with this serotype exhibited a higher requirement for bronchoscopic interventions and immunoglobulin therapy. These findings suggest that HAdV-7 infections should be incorporated into pediatric severe case warning systems, prioritizing the allocation of intensive care resources for such cases (Lin et al., 2017; Fu et al., 2019; Zhao et al., 2020; Liu et al., 2022).

We identified two HAdV-C suspected recombinant samples but found no evidence of recombination in the B/E subgroups. Previous studies have shown that HAdV-C establishes persistent infections after primary infection, characterized by intermittent shedding in the host (Alkhalaf et al., 2013). Such persistence creates conditions conducive to mixed infections with different serotypes within this subgroup, thereby promoting homologous recombination between distinct viral types (Wang et al., 2024). Notably, new recombinant HAdV strains may exhibit significant alterations in pathogenicity, tissue tropism, and clinical manifestations compared to their prototype strains (Wang et al., 2021; Wu et al., 2022), highlighting the importance of conducting continuous surveillance on emergent recombinant strains. Phylogenetic analysis revealed that all types exhibited complete segregation across the three gene fragments (Bootstrap >90%), except for HAdV-C in the Penton tree (Bootstrap <70%). The observed high heterozygosity in Hexon and Fiber gene sequences indicates that these proteins may be under significant immune pressure. The sequence diversity of the HAdV-C Penton gene is notably lower than that observed for HAdV-B and HAdV-E. However, Mao et al. ‘s (Mao et al., 2019) research demonstrated that the HAdV-C Penton phylogenetic tree could be divided into distinct clades with robust bootstrap support, implying a greater degree of genetic diversity in the Penton gene than previously recognized.

High nucleotide consistency across HAdV types was observed, clustering with strains circulating widely in China, reflecting the slow evolutionary rate of this DNA virus—a positive factor for vaccine development. Among the 14 HAdV-3 isolates analyzed, all exhibited >98.0% nucleotide identity across three core structural genes relative to the prototype strain. This is attributed to the combined effects of HAdV DNA polymerase’s high-fidelity replication, which enables HAdV-3 to maintain genomic stability for at least 50 years (Mahadevan et al., 2010). Notably, The 7 HAdV-7 strains isolated in this study exhibited low nucleotide consistency in the Hexon gene fragment, which comprises 28 sites of amino acid mutation. Research has shown that Hexon gene fragments contain specific antigenic epitopes that can stimulate the production of neutralizing antibodies in the host. Mutations in this region may result in immune escape (Lee et al., 2025). While genomic variation in HAdV-7 shows a high correlation with enhanced pathogenicity, establishing the causative relationship between specific mutation sites and the high-virulence phenotype observed in contemporary pediatric HAdV-7 strains requires further experimental data support. There are few reports of sporadic infections with HAdV-14. The singular HAdV-14 isolate showed >99.0% genomic conservation across all three genes compared to the prototype strain, and the fiber fragment contained three amino acid deletions. Studies have indicated that the fiber fragment contains type-specific antigenic epitopes that determine viral tropism, and that mutations in this region may result in different tissue tropism (Wang et al., 2014). In the HAdV-21 cohort (*n*=23), all except TJ2024–749 exhibited complete Penton sequence identity with GZ2019 (MW091531.1), classifying them as 21a subvariants. Due to the presence of 15 consecutive amino acid deletion variants on the Penton fragment (residues 287-301), the amino acid consistency is low, which may affect the RGD motif mediated viral endocytosis function on Penton. This deletion results in a truncated RGD loop, exhibiting structural homology to the naturally shorter loops found in HAdV-B3 and HAdV-B7. Research suggests that this conserved deletion pattern may compromise viral internalization mediated by the RGD motif and potentially contribute to the severe lower respiratory tract tropism associated with 21a infections (Hage et al., 2014).

Although our investigation provided crucial molecular insights into the epidemiological and clinical characteristics of HAdV infections in Tianjin, China, several limitations warrant consideration. Firstly, focusing solely on hospitalized pediatric ARIs patients may introduce selection bias, potentially underestimating HAdV-C prevalence (milder cases) and skewing subtype analysis towards more severe variants like HAdV-B7. Secondly, clinical manifestations could be influenced by co-infections. Thirdly, cost constraints limited Sanger sequencing to 100 randomly selected HAdV-positive samples, reducing statistical power for nucleotide diversity analysis of some serotypes. Fourthly, recombination analysis is based on partial gene fragments (Penton, Hexon, Fiber), and there may be additional recombination events in the unanalyzed regions, which may affect the precise localization of recombination breakpoints. Finally, sampling from a single institution may limit generalizability to the broader Tianjin population. These factors may lead to underestimation of HAdV prevalence and incomplete characterization of type distribution.

This two-year study systematically characterized HAdV epidemiology among pediatric inpatients with ARIs in Tianjin, revealing key insights into prevalence, age-specific susceptibility, seasonal dynamics, and evolving genotype distributions. Significant heterogeneity in genotype distributions across clinical phenotypes was observed. These findings are crucial for optimizing diagnosis, antiviral stewardship, and region-specific vaccine development.

## Data Availability

The datasets presented in this study can be found in online repositories. The names of the repository/repositories and accession number(s) can be found below: https://www.ncbi.nlm.nih.gov/, PV106134-PV106159.
