# Supplementary figures and images for: Epidemiological, clinical, and molecular analysis of human adenovirus infections in hospitalized children with acute respiratory infections in Tianjin, China
Source: Front Cell Infect Microbiol. 2025 Jul 14;15:1600990. doi: 10.3389/fcimb.2025.1600990 (PMC12301308; doi:10.3389/fcimb.2025.1600990)

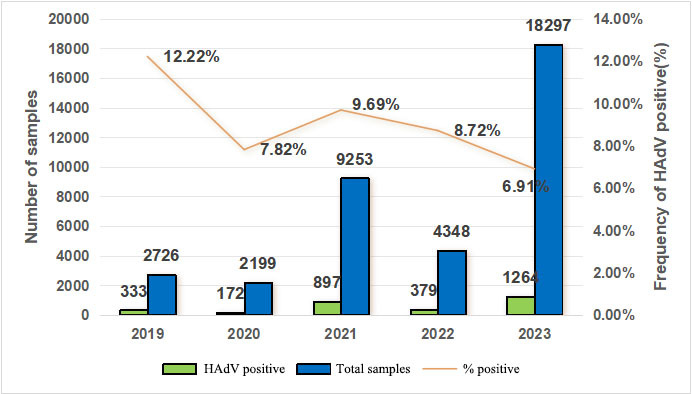

Supplement: Supplementary file 1 [file Image1.jpeg]
